# Increased frequency of dislocation following total hip arthroplasty for trauma: an age- and sex-matched cohort study

**DOI:** 10.1302/2633-1462.711.BJO-2026-0156.R1

**Published:** 2026-11-11

**Authors:** Thomas R. Williamson, Rabia Ali, Nicholas Kane, R. M. Dominic Meek†, Mark R. J. Jenkinson

**Affiliations:** 1 Department of Trauma and Orthopaedics, Queen Elizabeth University Hospital, Glasgow, UK; 2 School of Medicine, Dentistry and Nursing, University of Glasgow, Glasgow, UK

**Keywords:** Hip, Arthroplasty, Neck of femur, Osteoarthritis, Outcomes, total hip arthroplasty (THA), trauma, fractured neck of femur, periprosthetic fracture, osteoarthritis (OA), Anesthesiologists, comorbidities, dual-mobility implants, early dislocation, BMI

## Abstract

**Aims:**

The purpose of this study was to compare surgical techniques and postoperative outcomes in an age- and sex-matched cohort of patients undergoing total hip arthroplasty (THA) for neck of femur fracture (#NOF) and for osteoarthritis (OA).

**Methods:**

A single-centre, consecutive series of patients at minimum one-year follow-up following THA for #NOF were compared with a 1:1 age- and sex-matched cohort of patients following THA for osteoarthritis from the same institution. The primary outcome measure was frequency of dislocation at one year postoperatively. Secondary outcomes included rates of all-cause revision, infection, periprosthetic fracture, and mortality at one year and medium-term postoperatively. Cox regression compared medium-term outcomes between groups, accounting for unequal follow-up duration.

**Results:**

A total of 722 patients were included, with 361 in each group. BMI was greater in the OA group (28.9 kg/m^2^ vs 24.7 kg/m^2^; p < 0.001). Sex, age, American Society of Anesthesiologists grade, and comorbidity profile did not differ between groups (p > 0.247). Overall, 151 patients (41.8%) in the #NOF group received dual-mobility implants, compared with eight (2.2%) in the OA group (p < 0.001). Femoral offset was similar between groups (42.6 mm and 42.7 mm, p = 0.892). Dislocation rates were greater in the #NOF group than the OA group at one-year (4.4% vs 1.1%; p = 0.011) and by final follow-up. The one-year rates of periprosthetic fracture, all-cause revision, and mortality were similar between groups (p > 0.340), although risk of mortality was greater by final follow-up after THA for #NOF (hazard ratio 1.577; p = 0.033).

**Conclusion:**

Despite the more frequent use of dual-mobility implants in THA for #NOF than OA and the otherwise comparable patient demographics and implants used, the risk of early dislocation after THA for #NOF was four times greater than in an age- and sex-matched elective THA cohort. Early postoperative complication rates were otherwise comparable between the groups.

Cite this article: *Bone Jt Open* 2026;7(11):1455–1461.

## Introduction

The burden of hip disease worldwide is considerable, with osteoarthritis (OA) associated with substantial reductions in quality of life and increased mortality,^1,2^ and neck of femur fractures (#NOF) associated with profound disability and increased mortality.^3^

Total hip arthroplasty (THA) is the gold standard treatment for end-stage hip OA, providing excellent functional and quality-of-life outcomes for patients decades after surgery.^4^ THA is also increasingly used for patients with a displaced intracapsular #NOF and higher functional demands.^5,6^ While hip hemiarthroplasty (HA) has typically been the standard of care in this setting, THA offers greater postoperative function and patient satisfaction than HA following #NOF in appropriately selected patients.^7^ However, although the outcomes following THA in the elective setting are largely predictable and have been widely explored,^8^ it remains less clear whether patients undergoing THA for #NOF achieve comparable results.

Registry data have previously been used to compare outcomes between THA for OA and #NOF while adjusting for age and sex. Analysis of the Norwegian Arthroplasty registry found that THA for #NOF was associated with a 1.6-times greater risk of dislocation compared with THA for OA.^9^ Furthermore, multiple analyses of the National Surgery Quality Improvement Programme (NSQIP) database have found that THA for #NOF is associated with increased length of stay, increased operating time, and greater frequency of early medical complications and readmission after adjustment for age, sex, and BMI.^10,11^

However, registry data are typically limited by an inability to account for implant positioning, and some registry datasets may overlook important postoperative non-revision complications, such as dislocation and periprosthetic fracture. Surgical approach can affect complication rates such as dislocation rate.^12^ However, THA survivorship rates between approaches are largely comparable, and it has been suggested that surgeons should select their approach based on their familiarity and experience with each individual approach.^13^ The importance of implant positioning and surgical technique in this setting was highlighted by Verhagen et al,^14^ who reported no differences in frequency of revision or dislocation between trauma and elective THA in the context of satisfactory implant positioning by fellowship-trained arthroplasty surgeons. However, these findings are limited by their use of different approaches for trauma and elective THA, with anterior approaches preferred in the elective setting, and posterior or lateral in trauma.^14^ Furthermore, dual-mobility (DM) implants were not employed, despite their growing use in clinical practice and proposed reduction in dislocation risk in THA for both OA and #NOF.^15-17^

Despite the analyses from large registry series, there remains a paucity of evidence directly comparing surgical techniques, implant positioning, and clinical outcomes for THA for #NOF with that for OA, while accommodating for the differing demographic characteristics of the two groups.

The purpose of this study was to compare surgical techniques and postoperative outcomes at one year and final follow-up in an age- and sex-matched cohort of patients undergoing THA for #NOF or for OA.

## Methods

### Study design and population

Local institutional approvals were sought prior to study conduct. A retrospective analysis of a consecutive series of patients undergoing THA for fractured neck of femur (#NOF group) between July 2015 and July 2024 at a UK major trauma centre (Queen Elizabeth University Hospital, Glasgow) was performed. Propensity-score matching was used to identify a 1:1 age- and sex-matched cohort of patients who had undergone elective THA for primary OA (OA group) from the same centre. Patients with pathological femoral fractures were excluded from this study. Baseline demographic data, injury characteristics, surgical technique, and implant characteristics were collected from medical records.

### Outcome measures

The primary outcome measure was frequency of dislocation at one year postoperatively. Secondary outcomes included intraoperative blood loss and rates of all-cause revision, infection, periprosthetic fracture, and mortality at final follow-up and at one year postoperatively. Time to dislocation and mortality was recorded.

### Radiological assessment

Acetabular component anteversion and inclination angle were calculated from day one postoperative radiographs. Anteversion was assessed using cross-table lateral radiographs,^18^ while inclination was determined using the angle between the inter-ischial line and a line passing through the long axis of the acetabular component.^19^

### Surgical procedure

All patients received antibiotics and tranexamic acid at induction and were positioned laterally. The decision to use spinal or general anaesthesia was made at the anaesthetist’s discretion. A posterior approach was employed for all in the OA group, and all but two patients in the #NOF group (in whom a modified Hardinge approach was used). A consistent surgical technique was used for all patients undergoing THA through the posterior approach regardless of indication. Acetabular components were inserted with 40^o^ inclination and 20^o^ anteversion, and femoral components neutral to the native femoral neck. A complete posterior repair of both capsule and short external rotators was performed. The most common hip systems used were Exeter (Stryker, USA; 631 patients) and Corail (Depuy Synthes, USA; 41 patients). Where DM was employed at surgeons’ discretion, the modular dual-mobility (Stryker) system was employed in all cases. Patients underwent standardized postoperative care, including physiotherapy regimen, and were encouraged to weightbear without restriction, and all patients received at least four weeks’ chemical venous thromboembolism prophylaxis.

### Statistical analysis

Statistical analysis was performed using IBM SPSS Statistics v. 29.0.2.0 (IBM, USA). All continuous data were assessed for normality using Kolmogorov-Smirnov testing, and normally distributed data were reported with mean (SD). Non-normal data were reported using median and IQR. Continuous data were compared between groups using independent samples *t*-tests, or Mann-Whitney U tests for non-parametric data. Chi-squared was used for dichotomous data, with Fishers exact test preferred if the cell count was five or less. A Bonferroni correction was applied to accommodate multiple analyses. Cox proportional hazards modelling was used to compare outcomes between OA and #NOF groups over the full duration of follow-up, with group included as a covariate. Associated hazard ratios (HRs) and 95% CI were presented. Kaplan-Meier survival curves were created for time to dislocation, stratified by group, and censored at one year, and for mortality at final follow-up. Post hoc power analysis for one-year dislocation rate between groups revealed a power of 0.775 (G*Power). For one-year and medium-term dichotomous outcomes, odds ratios and 95% CIs were calculated. A p-value < 0.05 was considered significant for all analyses.

## Results

A total of 722 patients were included (361 in the #NOF group (69% female; mean age 67.6 years (SD 10.0)) and 361 in the OA group (69% female; mean age 67.5 years (SD 10.7)). One-year postoperative radiological and clinical data were available for 702 patients (97.2%). The mean overall follow-up was 5.9 years (1 to 9.4; SD 2.3) for the OA group and 4.8 years ( 1.1 to 9.3; SD 2.2) for the #NOF group (p < 0.001).

BMI was greater in the OA group than the #NOF group (28.9 kg/m^2^ and 24.7 kg/m^2^, respectively; p < 0.001). More patients were active- or ex-smokers in the #NOF group (p < 0.001), and the median deprivation index (SIMD) was lower (p = 0.007). American Society of Anesthesiologists (ASA) grade,^20^ Charlson Comorbidity Index,^21^ and prevalence of diabetes mellitus and immunosuppression did not significantly differ between the groups (Table I). In all, 720 patients (99.8%) underwent THA using a posterior approach, with two patients (0.2%) in the #NOF group undergoing THA using a modified Hardinge approach.

**Table I.** Demographic variables for osteoarthritis (OA) and femoral neck fracture (#NOF) groups.

Variable	OA (n = 361)	#NOF (n = 361)	p-value
Mean age, yrs (SD)	67.5 (10.7)	67.6 (10.0)	0.942
Female sex, n (%)	249 (69)	249 (69)	1.000
Mean BMI, kg/m^2^ (SD)	28.9 ( 5.4)	24.7 (4.8)	< 0.001*
Median SIMD, decile (IQR)	6 (3 to 9)	5 (2 to 9)	0.007*
Median CCI, units (IQR)	3 (2 to 4)	3 (2 to 4)	0.653
Dementia, n (%)	0 (0)	0 (0)	1.000
**ASA grade, n (%)**			0.247
1	58 (16.1)	45 (12.5)	
2	216 (59.8)	238 (65.9)	
3	84 (23.3)	72 (19.9)	
4	3 (0.8)	6 (1.7)	
Immunosuppression, n (%)	18 (5)	24 (6.6)	0.340
Diabetes mellitus, n (%)	37 (10.2)	37 (10.2)	1.000
**Smoker, n (%)**			< 0.001*
Active	38 (10.5)	78 (21.6)	
Ex	69 (19.1)	94 (26)	
Never	254 (70.4)	189 (52.4)	

* Indicates significance at p < 0.05.

ASA, American Society of Anesthesiologists; CCI, Charlston Comorbidity Index; SIMD, Scottish Index of Multiple Deprivation.

### Implant usage & positioning

Postoperative combined femoral offset was similar between #NOF and OA groups (42.6 mm (SD 4.5) and 42.7 mm (SD 4.5); p = 0.892). A total of 151 (41.8%) patients in the #NOF group received DM implants, compared with eight (2.2%) in the OA group (p < 0.001). In all, 526 (72.9%) acetabular components were inserted with both 20° (± 10°) anteversion and 40^o^ (± 10°) inclination,^14^ which did not differ between #NOF and OA groups (p = 0.357).

Bearing and cementing techniques significantly differed between groups, with hybrid arthroplasty and metal-on-polyethylene bearings used more frequently in the #NOF than the OA group (p < 0.001) (Table II).

**Table II.** Implant-specific variables used in osteoarthritis (OA) and femoral neck fracture (#NOF) groups.

Variable	OA (n = 361)	#NOF (n = 361)	p-value
**Cementing (n, %)**			< 0.001*
Cementing	96 (26.6)	47 (13.0)	
Hybrid	224 (62.0)	289 (80.1)	
Uncemented	41 (11.4)	25 (6.9)	
Mean cup size, mm (SD)	51.9 (3.9)	51.3 (3.5)	0.033*
Mean head size, mm (SD)	32.3 (2.9)	35.9 (5.5)	< 0.001*
Mean head size (unipolar only), mm (SD)	32.1 (2.4)	32.0 (2.4)	0.654
Mean combined offset, mm (SD)	42.7 (4.5)	42.6 (4.5)	0.892
Dual-mobility, n (%)	8 (2.2)	151 (41.8)	< 0.001*
**Bearing combination, n (%)**			< 0.001*
Metal-on-polyethylene	201 (55.7)	249 (69)	
Ceramic-on-polyethylene	104 (28.8)	97 (26.9)	
Ceramic-on-ceramic	56 (15.5)	15 (4.2)	
Mean cup inclination angle, ^o^ (SD)	41.1 (7.2)	41.6 (6.9)	0.340
Mean cup version, ^o^ (SD)	22.1 (6.3)	23.3 (6.7)	0.013*

* Indicates significance at p < 0.05.

### One-year clinical outcomes

The one-year dislocation rates were significantly greater in the #NOF group (4.4%) than the OA group (1.1%; HR 4.1 (1.36 to 12.19); p = 0.012). The median time to dislocation was not significantly earlier in the #NOF group than the OA group (24 days (IQR 8 to 69) and 66 days (IQR 15 to 135), respectively; p = 0.290) (Figure 1).

**Fig. 1:**
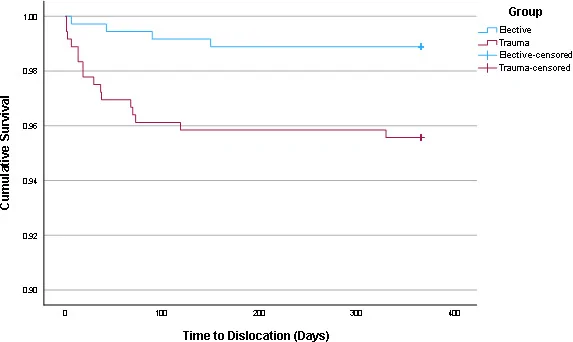
Kaplan-Meier survival analysis comparing dislocation frequency for femoral neck fracture and osteoarthritis groups by one-year postoperatively.

Rates of periprosthetic fracture (0.8% and 0.3%, respectively; p = 0.624), all-cause revision surgery (1.9% and 0.8%, respectively; p = 0.340), and mortality (1.9% and 1.1%, respectively; p = 0.546) were similar between groups (Figure 2). Mean time to death was 201 days (SD 119) for the #NOF group and 244 days (SD 85) for the OA group (p = 0.540), with one death within 30 days postoperatively in the #NOF group. Intraoperative blood loss did not significantly differ between groups (345 ml (SD 223) for the #NOF group, and 328 ml (SD 139) for the OA group; p = 0.222).

**Fig. 2:**
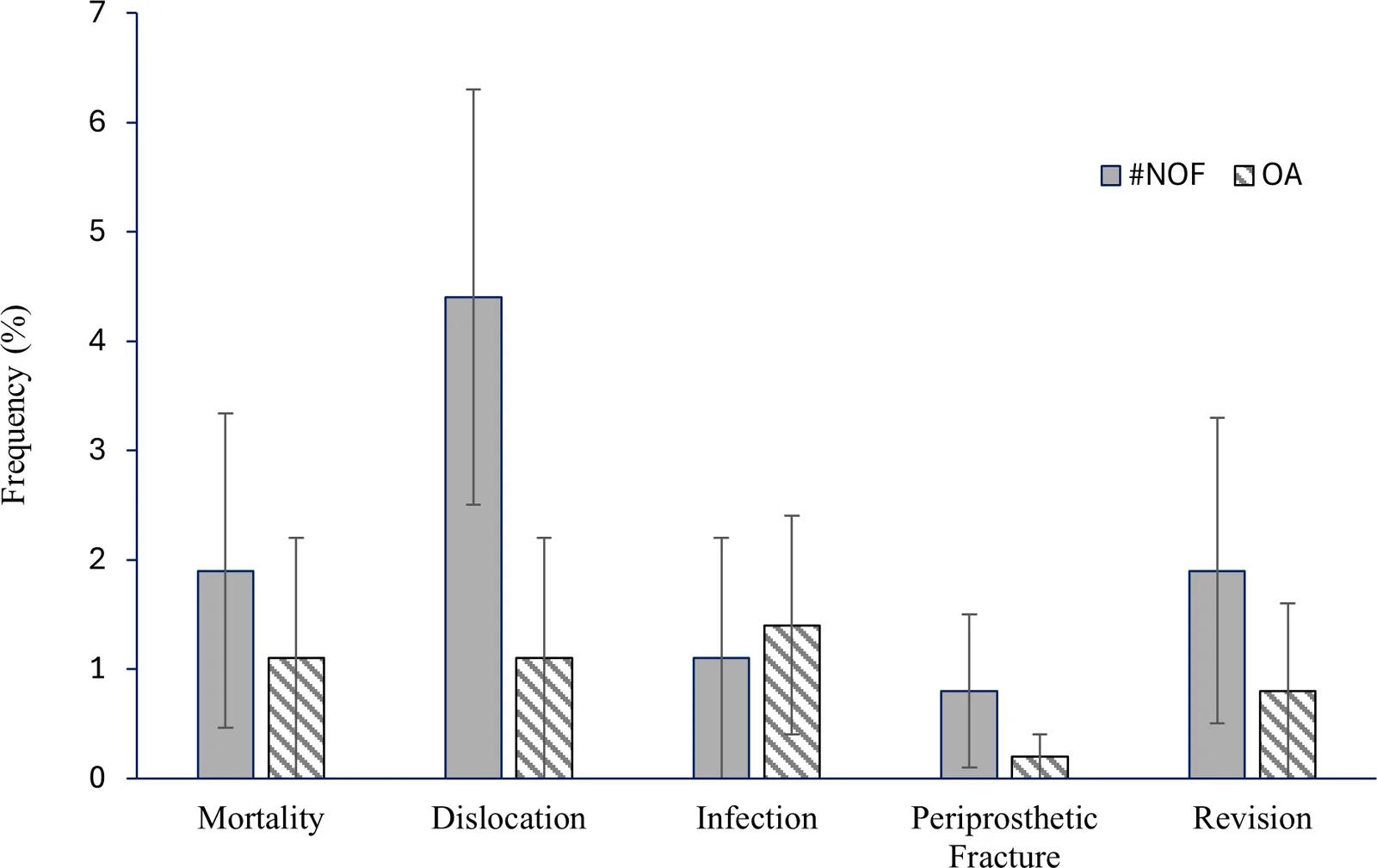
Prevalence of adverse outcomes at one-year postoperatively for patients undergoing total hip arthroplasty for femoral neck fracture (#NOF) and osteoarthritis (OA).

### Final follow-up clinical outcomes

Accounting for unequal follow-up duration, mortality risk was significantly greater in the #NOF group (HR 1.577, 95% CI 1.038 to 2.395; p = 0.033) (Figure 3). Risk of dislocation was significantly greater in the #NOF group (HR 3.936, 95% CI 1.582 to 9.794; p < 0.001). No differences were observed between groups for risk of periprosthetic fracture (p = 0.107) or revision arthroplasty (p = 0.496). Five patients underwent revision arthroplasty for recurrent dislocation, of which four were in the #NOF group (1.1%) and one was in the OA group (0.3%; p = 0.156).

**Fig. 3:**
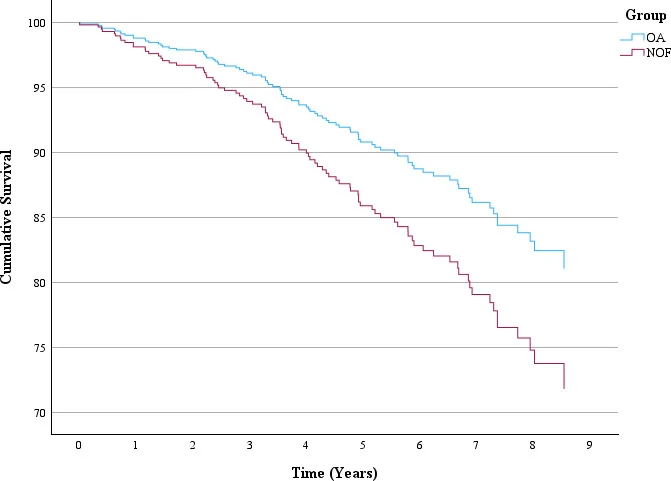
Cox regression model comparing mortality between osteoarthritis (OA) and femoral neck fracture (#NOF) groups.

## Discussion

This study shows that despite appropriate implant positioning, patients undergoing THA for #NOF experience early dislocation four times more frequently than those of comparable age and sex undergoing elective THA. Surgeons’ decisions regarding implant choices also differ in this group, with a high frequency of DM use, and a preference towards hybrid techniques and metal-on-polyethylene implants.

The increased frequency of early dislocation observed in this study for patients with #NOF compared with OA is in keeping with that reported in multiple registry analyses.^9^ This series included all THA for #NOF from a UK major trauma centre over a nine-year period, allowing broader generalization to UK clinical practice, including the pragmatic use of DM constructs. However, Verhaegen et al^14^ proposed that the increased risk of dislocation following THA for #NOF may be a product of surgical technique, as they observed comparable dislocation rates with elective THA when both were performed by an arthroplasty surgeon. Interestingly, the excellent radiological outcomes reported by Verhaegen et al^14^ in their series are comparable with those reported in this study, but the dislocation rates observed differ considerably, despite their use of traditional non-DM components. The preferential use of the anterior approach in OA cases may contribute to this, but dislocation in THA is multifactorial in nature, and this difference may also be driven by patients’ individual comorbidities and risk factors for dislocation, that are largely unknown from their series. Nonetheless, the current study employed a posterior approach for 99.7% of THAs performed, standardizing this aspect of the comparison and mitigating confounding, yet still found the risk of dislocation to be greater for the #NOF group than the OA group.

Data from both the German Arthroplasty Registry and the American College of Surgeons NSQIP suggest that the mortality rate associated with THA #NOF may be between four to six times greater than that for OA, despite matching for age and sex.^22,23^ Multiple variables, including BMI and comorbidities, have been cited as factors influencing this trend.^24^ However, a smaller series assessing nonagenarians reported comparable mortality, revision, and periprosthetic fracture rates between age- and sex-matched OA and #NOF patients, despite their advanced age.^25^ Similarly, we found that when controlling for age and sex, the one-year mortality, infection, all-cause revision, and periprosthetic fracture rate was comparable between groups, although mortality by final follow-up was greater in the #NOF group. While BMI was greater in the #NOF group than the OA group in this study, comorbidity score, ASA grade, and the prevalence of immunosuppression and diabetes mellitus did not differ between groups, which may account for the different findings in this study compared with the registry analyses.

This study also showed that surgeons preferences in the setting of #NOF were for hybrid, metal-on-polyethylene THA, with regular use of DM implants. Studies have typically failed to identify any difference in clinical outcomes following THA for #NOF between cemented or hybrid arthroplasty.^26^ However, there is a growing interest in the role of DM implants in the potential reduction of dislocation for patients with #NOF. DM constructs have two articulating surfaces and so have been proposed to provide an increase in hip stability due to the increased jump distance.^27^ Clinical studies have suggested than DM is associated with reduced risk of revision and dislocation in THA for #NOF, although concern has been raised regarding longevity due to increased wear profiles, and the potential for intraprosthetic dislocation.^16,28^ This study shows a clear preference from surgeons to use DM constructs in THA for #NOF, but further analysis of the specific role of DM components and implant combinations is required to determine whether these implants can safely mitigate against the increased risk of dislocation in this population, and prospective randomized controlled trials have been proposed to assess this further.^29^

This study is primarily limited by its retrospective nature, although this was mitigated by the minimal loss to follow-up (2.8%). The one-year follow-up employed in this study is appropriate to assess early postoperative outcomes, but longer-term follow-up would be beneficial given that THA for #NOF is performed in patients anticipated to expect years of independence postoperatively. The one-year dislocation rate was chosen as the primary outcome measure to standardize comparison between groups, as follow-up data beyond one year for #NOF THA was shorter than that available for the OA group, limiting comparisons. While this study shows a clear difference in early postoperative risk profiles for patients undergoing THA for #NOF compared with OA, PROMs may have helped to further assess any subtle differences in function or quality of life less readily appreciated in the current analysis. Assessment of acetabular implant positioning was performed using plain radiographs rather than cross-sectional imaging, which may not provide as precise or reliable an assessment of cup position, particularly when accounting for pelvic tilt. Furthermore, femoral component version was not able to be reliably assessed, and so combined version remains unknown, which is established to impact hip stability postoperatively. Patient suitability for THA for #NOF was determined after multidisciplinary assessment and input from at least three consultant trauma and orthopaedic surgeons, including at least one fellowship-trained hip arthroplasty surgeon, which introduces the risk of selection bias in this cohort. Given that implant usage was at the discretion of the operating surgeon, DM components were preferentially employed in THA for #NOF rather than OA, and so this study may underestimate the comparative risk of dislocation after THA for #NOF when comparable acetabular bearings are employed. Further exploratory analysis of factors predictive of adverse outcomes in both groups may help surgical planning, although this was outwith the scope of the current study.

In conclusion, DM implants are more commonly used in THA for #NOF compared with OA. Despite the largely comparable patient demographics, implant positioning and usage, this study found the risk of early dislocation after THA for #NOF to be four times greater than in an age- and sex-matched elective THA cohort in a UK Major Trauma Centre. The one-year postoperative complication rates were otherwise comparable between the groups. Surgeons should be mindful of this in preoperative patient counselling.

**Take home message**

- This study found that despite the more frequent use of dual-mobility implants in total hip arthroplasty (THA) for neck of femur fracture (#NOF) than osteoarthritis and the otherwise comparable patient demographics and implants used, the risk of early dislocation after THA for #NOF was four-times greater than in an age- and sex-matched elective THA cohort.

- Early postoperative complication rates were otherwise comparable between the groups.

## Data Availability

The datasets generated and analyzed in the current study are not publicly available due to data protection regulations. Access to data is limited to the researchers who have obtained permission for data processing. Further inquiries can be made to the corresponding author.
